# Recommendations on visit duration and sample number requirements for an automated head chamber system

**DOI:** 10.1093/jas/skae158

**Published:** 2024-06-04

**Authors:** Matthew R Beck, Logan R Thompson, Jarret A Proctor, R Ryan Reuter, Stacey A Gunter

**Affiliations:** USDA-ARS, Livestock Nutrient Management Research Unit, Bushland, TX 79012, USA; Department of Animal Sciences and Industry, Kansas State University, Manhattan, KS 66506, USA; Department of Animal Science, Texas A&M University, College Station, TX 77843, USA; Department of Animal and Food Science, Oklahoma State University, Stillwater, OK 74078, USA; USDA-ARS, Oklahoma and Central Plains Agricultural Research Center, El Reno, OK 73036, USA

**Keywords:** beef cattle, feedlot, gas flux, pasture, ruminants

## Abstract

Automated head chamber systems (AHCS; GreenFeed, C-Lock Inc., Rapid City, SD) increasingly are being used for measuring the gas flux of unrestrained cattle. There are a wide range of recommendations for what constitutes a “good” visit (i.e., duration) to an AHCS and how many visits are required for the AHCS to quantify gas fluxes accurately and precisely. Accordingly, the purpose of this experiment was to investigate the effects of visit duration thresholds and the subsequent effects of these thresholds on the number of visits needed to provide adequate estimates of carbon dioxide (CO_2_) and methane (CH_4_) emissions, and oxygen (O_2_) consumption by beef cattle. This analysis utilized data from three previously published experiments with grazing beef steers and one experiment with finishing beef steers, with 103 steers total. When comparing all available visits, there was excellent agreement [Lin’s concordance correlation coefficient (CCC) ≥ 0.96] between visits ≥ 3 min in duration and those ≥ 2 min for the three gases in all four experiments. When data from all four experiments were pooled, there was excellent agreement between visits ≥ 3 min and those ≥ 2 min and ≥ 1 min for all gases (CCC ≥ 0.96). These results suggest that estimates from visits ≥ 2 min are like those from visits ≥ 3 min. Next, we investigated if including visits ≥ 2 min or ≥ 1 min would increase the minimal number of visits required to provide excellent agreement with the “gold-standard” (mean of all visits ≥ 3 min). For this, we used only one of the experiments and randomly selected visits per animal ranging from *n* = 5 to 60, in increments of 5. The sole experiment was used because all animals had more than 60 visits. We then assessed the agreement between the “gold-standard” (mean of all visits ≥ 3 min [144 ± 55.01 visits per steer]) estimates of CO_2_, O_2_, and CH_4_. The minimum number of visits required to achieve excellent agreement (CCC ≥ 0.90) to the “gold-standard” estimate for all gases was 30 visits ≥ 3 min in duration, or 40 visits ≥ 2 min in duration. Visits ≥ 1 min in duration did not achieve excellent agreement, even when 60 were used. Based on these results, we recommend excluding visits < 3 min in duration with 30 minimum visit records per animal. However, if researchers choose to implement a 2-min visit duration threshold then 40 visit records are needed per animal.

## Teaser

When using the automated head chamber system (GreenFeed, C-Lock Inc., Rapid City, SD) to estimate enteric methane emissions, it is recommended to use a 3-min minimum visit duration and 30 visit records per animal. If a researcher chooses to use a 2-min minimum visit duration, then 40 visit records per animal are recommended.

## Introduction

Measuring the gas flux of unrestrained cattle is becoming increasingly important. Enteric methane (**CH**_**4**_) is an important greenhouse gas to be mitigated as CH_4_ mitigation represents the most promising means to address climate change in a short timeframe (Ocko et al., 2021; Beck et al., 2022). Furthermore, quantification of CH_4_ and carbon dioxide (**CO**_**2**_) emissions, and oxygen (**O**_**2**_) consumption provide a means to conduct energetic studies through indirect calorimetry (Kleiber, 1935) by calculating energy expenditure (Brouwer, 1965; Kaufmann et al., 2011). Accordingly, it is becoming increasingly important to assess CH_4_ and CO_2_ emissions and O_2_ consumption of cattle. Researchers are increasingly employing automated head chamber systems (**AHCS**; GreenFeed, C-Lock Inc., Rapid City, SD; Gunter and Beck, 2018) to evaluate gas flux of unrestrained cattle. The AHCS estimates daily gas flux of cattle by using the average of multiple spot samples, collected while the animals are visiting the units. The rapid adoption of this technology necessitates the uniformity of sampling and data preprocessing protocols.

There currently exists a range of recommendations of what constitutes an acceptable visit to the AHCS and how many visits are necessary to achieve accurate and precise data. For example, C-Lock Inc. currently recommends using data from visits that are ≥ 2 min in duration, while Arthur et al. (2017) recommended using visits ≥ 3 min in duration. Furthermore, Arthur et al. (2017) recommended a minimum of 30 visits to achieve adequate estimates of CH_4_, while Dressler et al. (2023) recommended 40 visits per animals to achieve adequate estimates of CO_2_, O_2_, and CH_4_. One key difference between these experiments, besides the difference in production setting, was that Arthur et al. (2017) assessed visits that were ≥ 3 min, whereas Dressler et al. (2023) only considered visits that were ≥ 2 min. Arthur et al. (2017) determined that a minimum 2-min visit duration required 45 visits, while a 3-min minimum visit duration only required 30 visits. Accordingly, the difference in recommendations for required visits between Dressler et al. (2023) and Arthur et al. (2017) are likely due to differences in visit duration minimums. To the best of our knowledge, the relationship between minimum visit duration and the number of required visits has only been explored for CH_4_ and CO_2_ emissions by Arthur et al. (2017) and has not been investigated for O_2_ consumption. Therefore, the current experiment had three main objectives. The first was to determine the effects of using different visit duration thresholds on estimates of CO_2_, O_2_, and CH_4_. The second objective was to explore how the estimates of CO_2_, O_2_, and CH_4_ differ with increasing visit duration lengths. The final objective was to determine how these different visit duration thresholds influence the number of visits required to achieve adequate estimates of CO_2_, O_2_, and CH_4_ flux.

## Materials and Methods

### Compiled dataset

Data from four, previously published experiments were compiled (Beck et al., 2018, 2019; Thompson et al., 2019; Proctor, 2023). All procedures involved with animal handling and care are described in the referenced manuscripts and were approved by an Institutional Animal Care and Use Committee (IACUC) prior to trial start. Beck et al. (2018), Beck et al. (2019), and Thompson et al. (2019) were approved by the Oklahoma State University IACUC (#AG-16-19). Proctor (2023) was approved by the West Texas A&M University IACUC (#2022.01.002). These experiments all employed AHCS equipped with a CO_2_ and CH_4_ analyzer, and a paramagnetic O_2_ analyzer. Beck et al. (2018) investigated different amounts of whole-cotton seed supplementation to steers (*n* = 13; initial body weight [BW] = 317 ± 23 kg) grazing tall-grass prairie pastures. In Beck et al. (2019) steers (*n* = 20; initial BW = 269 ± 35 kg) were used to test the ability of three different fat supplements to influence CH_4_ emissions while grazing tall-grass prairie pastures. Thompson et al. (2019) utilized eight steers (initial BW = 262 ± 33 kg) and eight heifers (BW = 240 ± 21 kg) grazing winter wheat pasture to assess the impacts of an energy supplementation on CH_4_ emissions. The study of Proctor (2023) collected dry matter intake, growth performance, and gas flux data from 53 cross-bred finishing beef steers (initial BW = 525 ± 29.8 kg). The AHCS were calibrated, and CO_2_ recoveries were assessed as recommended by C-Lock Inc. for all experiments.

Herein, an AHCS visit is defined as a spot sampling instance for when an animal is attracted to the AHCS by a pelleted bait supplement. During each visit, the AHCS used by Beck et al. (2018), Beck et al. (2019), and Thompson et al. (2019) was set to dispense six drops of pellets every 30 s and the AHCS used by Proctor (2023) was set for eight drops every 24 s. All individual visit data, including visits <2 min in duration, from these experiments were provided by C-Lock Inc. This data set included 31,039 visit observations. Any observations with airflow <26 L/s were excluded from the data frame, as suggested by Gunter et al. (2017) as airflow <26 L/s would result in incomplete capture of the animals’ breath clouds. This removed 6,665 observations. Furthermore, estimates of CO_2_ emissions greater than 20,000 g/d; of O_2_ consumption greater than 15,000 g/d; and CH_4_ emissions greater than 500 g/d were removed because these represent biologically infeasible estimates and were identified as obvious outliers upon visual inspection of a scatter graph of all AHCS visits across the experimental day. This removed another 179 observations, resulting in a final data frame of 24,195 individual visit observations. The removal of these 179 observations represents a conservative approach as they represent > 10 × the standard deviation (SD) plus the mean.

Gas flux estimates were then derived by taking the arithmetic mean of the individual visit observations. It has been suggested that diurnal variation of gas estimates should be accounted for to derive the most accurate estimates of gas flux. One means for diurnal variation to be controlled in an experiment utilizing the AHCS is by programming the unit with minimum durations between visits. This forces the animal to visit throughout the day by not dispensing feed when a visit is not allowed. The minimum duration between visits was set at 6 h for Beck et al. (2018) and 4 h for Beck et al. (2019), Thompson et al. (2019), and Proctor (2023). For all experiments, visit patterns followed a similar pattern to typical grazing patterns for pastoral cattle and meal patterns for pen-fed cattle (Supplementary Figure 1). In all experiments, there was a big peak of AHCS visits in the morning, another one in the afternoon, and then typically a smaller peak overnight. Another proposed means to control for diurnal variation in AHCS experiments is time-bin averaging (Manafiazar et al., 2017). This approach groups visits by time of day into bins, where bins are 000 to < 0300; 0300 to < 0600; 0600 to < 0900; 0900 to < 1200; 1200 to < 1500; 1500 to < 1800; 1800 to < 2100; and 2100 to < 2400. The within-time bin average is calculated and then the daily average is made across bins. This approach theoretically weights the gas flux estimate by time of day. We compared gas flux estimates using merely arithmetic averages across each visit with the time bin averaging approach for each experiment and across experiments. The mean difference between the two approaches were small for CH_4_ emissions (2.8 to 13.3 g/d; 1.6% to 6.4% of the bin average mean) for all four experiments (Supplementary Table 1). Comparing the two methods for data pooled across the four experiments yielded small mean differences for CH_4_ emissions (1.5 g/d; 0.9% of the bin average mean; Supplementary Table 1). These small differences are similar to the small mean differences reported by Manafiazar et al. (2017), who reported only 4.6 g/d difference between the arithmetic and time bin averaging methods. This likely indicates that diurnal variation was adequately accounted for by setting minimum allowable times between visits, thereby forcing the cattle to visit the AHCS at different times of the day.

One potential issue with utilizing the time-bin averaging technique is that it may over-inflate time-bins with relatively low numbers of visits. We know that as the number of observations that make up an average increases, the precision and accuracy of that estimate increases. Accordingly, if there are time-bins with low numbers of visits, by equally weighting that time-bin relative to other time-bins, the overall estimate precision and accuracy may be reduced. Based on these findings (Supplementary Table 1) and considerations, the remaining comparisons were conducted using simple arithmetic means.

### Description of comparisons

The current experiment aimed to conduct three comparisons. In all comparisons, the average of all visits ≥ 3 min was used as the “gold-standard”, as recommended by Arthur et al. (2017), and all other estimates were compared against this standard. The ≥ 3 min visit duration was selected as the “gold-standard” as it represents the most conservative visit duration threshold. For all comparisons, estimates were compared to the “gold-standard” for precision (Pearson’s correlation coefficient; **r**), accuracy (bias correction factor; **Cb**), agreement (Lin’s concordance correlation coefficient; **CCC**; Lin, 1989, 2000), and the root mean square error (RMSE) expressed as a percent of the ≥ 3 min estimate (**RMSE**). For all comparisons, only CCC values that were ≥ 0.90 were considered as excellent agreement.

For the first objective, we explored the effects of utilizing different visit duration thresholds (≥3, ≥2, ≥1 min, or all visits) on estimates. To do this, the average and SD of visit duration, number of visits per animal, CO_2_, O_2_, and CH_4_ estimates for each experiment for these four visit duration thresholds were calculated. The estimates from ≥ 2 min, ≥ 1 min, and all visits were compared to the ≥ 3 min estimate. For this comparison, data from all four experiments were used.

For the second objective, we aimed to determine how visit duration influences AHCS visits per animal, visit duration, CO_2_, O_2_, and CH_4_. The visit duration ranges included ≥ 3 min; < 1 min; observations ≥ 1 min and < 2 min; observations ≥ 2 min and < 3 min; observations ≥ 3 and < 4 min; observations ≥ 4 min and < 5; observations ≥ 5 and < 6; and observations ≥ 6 min. The mean and SD of these variables were calculated. Then the estimates of CO_2_, O_2_, and CH_4_ were compared to the “gold-standard” as described above. Again, the data from all four experiments were utilized for this comparison.

The final comparison explored in this experiment was to determine if different visit duration thresholds resulted in the need for increased number of visits to provide adequate estimates of CO_2_, O_2_, and CH_4_. To explore this, we selected visits with ≥ 3 min, ≥ 2 min, and ≥ 1 min visit durations. Next, visit numbers for each animal were randomly selected ranging from 5 to 60 increasing in increments of 5. For this comparison, only data from Proctor (2023) was utilized due to a lack of visits available from the other three experiments, where all animals had greater than 30 visits each, but many did not have more than 40 visits each. The estimates of CO_2_, O_2_, and CH_4_ from the different visit duration thresholds and the visit numbers were then compared, using the same statistics as described above, against the average of all visits ≥ 3 min.

### Statistical analysis

All data processing and analysis was conducted in R (v.4.3.1; R Core Team, 2023). Pearson’s correlation was calculated using the ‘cor.test’ function of base R. The Cb and CCC were determined using the ‘CCC’ function and RMSE using the ‘RMSE’ function of the ‘DescTools’ package (Signorell et al., 2023). Figures were generated using ‘ggplot2’ (Wickham, 2016).

## Results

Table 1 displays the descriptive statistics for each of the four experiments as affected by visit duration thresholds. As expected, the visit duration decreased, and the number of visits increased when the visit duration threshold became less conservative for all experiments. On average, going from visits ≥ 3 min to visits ≥ 2 min, to visits ≥ 1 min and all visits increased the number of visits per animal by 27.3%, 48.2%, and 117.2%, respectively, on average across the experiments. On average, estimates of CO_2_ and O_2_ were less than 1% different for ≥ 2 min and ≥ 1 min estimates compared with the ≥ 3 min estimate. Estimates of CH_4_ were less than 1% different for ≥ 2 min estimate compared with ≥ 3 min estimate. The ≥ 2 min CH_4_ estimate was −2.6% less than the ≥ 3 min estimate. The ≥ 3 min estimate was 5.5%, 9.6%, and 22.8% greater than the estimates of CO_2_, O_2_, and CH_4_ from all visits, respectively, on average across the four experiments.

**Table 1.** Descriptive statistics (arithmetic mean ± SD) of the experiments were included for analysis and descriptions of the use of the automated head chamber system (AHCS) and gas flux as influenced by visit duration thresholds (i.e., ≥ 3, ≥ 2, ≥ 1, or all visits)

	Visit duration thresholds			
Item^1^^,^^2^	≥3 min	≥2 min	≥1 min	All
*Beck 2018*				
Dur.	4.3 ± 0.44	4.0 ± 0.37	3.6 ± 0.35	2.6 ± 0.40
Visit av.	1.5 ± 0.15	1.7 ± 0.15	2.0 ± 0.18	3.0 ± 0.49
Visits	61.1 ± 16.02	77.6 ± 20.82	93.6 ± 24.51	150.4 ± 46.24
CO_2_	7,143.3 ± 564.93	7,173.8 ± 568.92	7,201.6 ± 548.76	6,891.5 ± 454.37
O_2_	5,230.6 ± 432.51	5,267.0 ± 437.75	5,288.3 ± 420.66	4,958.1 ± 403.55
CH_4_	198.3 ± 22.54	194.9 ± 22.63	191.4 ± 21.04	151.00 ± 17.47
*Beck 2019*				
Dur.	4.1 ± 0.39	3.8 ± 0.33	3.6 ± 0.38	2.9 ± 0.39
Visit av.	1.5 ± 0.25	1.6 ± 0.37	1.8 ± 0.47	2.5 ± 0.88
Visits	49.1 ± 17.63	64.1 ± 25.71	73.8 ± 31.48	106.9 ± 53.19
CO_2_	6,067.5 ± 638.43	6,104.8 ± 643.30	6,091.0 ± 638.39	5,918.4 ± 604.65
O_2_	4,461.6 ± 467.95	4,508.6 ± 475.26	4,508.8 ± 471.39	4,278.8 ± 464.70
CH_4_	176.0 ± 23.16	176.1 ± 24.67	174.7 ± 25.60	148.8 ± 22.56
*Thompson*				
Dur.	3.7 ± 0.15	3.4 ± 0.19	3.4 ± 0.19	3.0 ± 0.22
Visit av.	1.6 ± 0.26	1.9 ± 0.27	2.0 ± 0.27	2.4 ± 0.50
Visits	51.3 ± 12.22	67.4 ± 10.16	70.5 ± 10.30	85.8 ± 18.08
CO_2_	6,117.2 ± 387.38	6,118.5 ± 411.45	6,113.6 ± 404.05	5,983.9 ± 400.17
O_2_	4,390.3 ± 325.51	4,399.2 ± 336.34	4,398.7 ± 332.80	4,175.6 ± 329.42
CH_4_	169.5 ± 13.51	168.4 ± 13.52	167.0 ± 13.68	150.9 ± 15.03
*Proctor*				
Dur.	4.4 ± 0.56	4.1 ± 0.52	3.7 ± 0.54	2.7 ± 0.69
Visit av.	2.4 ± 0.58	2.7 ± 0.76	3.3 ± 1.12	4.9 ± 2.24
Visits	144.0 ± 55.01	173.3 ± 71.22	218.7 ± 104.70	342.0 ± 204.47
CO_2_	9,860.5 ± 835.81	9,688.8 ± 804.05	9,465.2 ± 799.51	8,673.7 ± 750.93
O_2_	6,706.4 ± 631.08	6,625.2 ± 653.94	6,439.9 ± 687.98	5,423.7 ± 732.61
CH_4_	149.9 ± 33.00	148.5 ± 34.51	142.6 ± 36.12	116.1 ± 37.84

^1^Beck 2018 = Beck et al. (2018); Beck 2019 = Beck et al. (2019); Thompson = Thompson et al. (2019); Proctor = Proctor (2023).

^2^Dur. = Visit duration, minutes; visit av. = average visits per d; visits = number of visits to the AHCS; CO_2_ = carbon dioxide, g/d; O_2_ = oxygen consumption, g/d; CH_4_ = methane emissions, g/d.

Table 2 presents comparative statistics between the ≥ 3 min visit duration estimates and the ≥ 2 min, ≥ 1 min, and all visit estimates for CO_2_, O_2_, and CH_4_ for each experiment and the pooled results. None of the experiments achieved the ≥ 0.90 threshold for CCC set for O_2_ and CH_4_ when all visits were included in the analysis. When all visits were included in the analysis for CO_2_, 2 experiments did not hit the threshold (Beck et al., 2018; Proctor, 2023), and two experiments did (Beck et al., 2019; Thompson et al., 2019). The Beck et al. (2019) and Thompson et al. (2019) achieved the threshold for CCC for all gases when ≥ 1 min visit durations were included in the analysis. For Beck et al. (2018), CO_2_ and O_2_ reached the threshold, but CH_4_ did not when ≥ 1 min visit durations were used. For Proctor (2023), CH_4_ reached the CCC threshold, but CO_2_ and O_2_ did not when ≥ 1 min visit durations were included. The CCC for the ≥ 2 min visit duration and ≥ 1 min visit duration was greater than the CCC threshold used for all gases (CCC ≥ 0.96) when all four experiments were pooled in the analysis. However, this threshold was not reached for any gas when all visits were included (CCC ≤ 0.86) for the pooled analysis.

**Table 2.** Comparative statistics of ≥ 3-min visit duration with ≥ 2 min., ≥ 1 min., or all visits (All) for methane emissions (CH_4_), carbon dioxide emissions (CO_2_), and oxygen consumption (O_2_) measured using an automated head chamber system (GreenFeed; C-Lock Inc., Rapid City, SD), by experiment

	CO_2_			O_2_			CH_4_		
Item^1^^,^^2^	≥2 min	≥1 min	All	≥2 min	≥1 min	All	≥2 min	≥1 min	All
*Beck 2018*									
r	0.98	0.99	0.96	0.99	0.99	0.89	0.97	0.94	0.89
Cb	1.00	0.99	0.86	1.00	0.99	0.81	0.99	0.95	0.24
CCC	0.98	0.98	0.83	0.98	0.98	0.72	0.96	0.89	0.22
RMSE	1.41	1.52	4.33	1.40	1.61	6.34	3.25	5.19	24.40
*Beck 2019*									
r	0.99	0.99	0.97	0.99	0.99	0.96	0.99	0.99	0.91
Cb	0.99	1.00	0.97	0.99	0.99	0.92	1.00	0.99	0.57
CCC	0.98	0.99	0.94	0.98	0.98	0.88	0.98	0.98	0.52
RMSE	1.80	1.55	3.46	2.05	2.00	5.11	2.37	2.70	16.38
*Thompson*									
r	0.98	0.99	0.96	0.99	0.99	0.93	0.96	0.96	0.80
Cb	1.00	1.00	0.94	1.00	1.00	0.81	1.00	0.98	0.52
CCC	0.98	0.98	0.90	0.99	0.99	0.76	0.96	0.94	0.42
RMSE	1.17	1.10	2.87	1.01	1.00	5.55	2.33	2.69	12.14
*Proctor*									
r	0.98	0.97	0.85	0.99	0.96	0.77	0.99	0.97	0.92
Cb	0.98	0.89	0.47	0.99	0.92	0.35	1.00	0.97	0.68
CCC	0.96	0.87	0.40	0.98	0.89	0.27	0.99	0.95	0.62
RMSE	2.32	4.52	12.82	1.86	4.81	20.36	3.41	7.35	24.68
*Pooled Exp.*									
r	1.00	1.00	0.98	1.00	0.99	0.86	0.99	0.98	0.90
Cb	1.00	0.99	0.88	1.00	0.99	0.72	1.00	0.98	0.68
CCC	1.00	0.98	0.86	1.00	0.98	0.62	0.99	0.96	0.61
RMSE	2.17	4.00	11.32	1.81	4.18	17.54	3.04	5.62	21.56

^1^Beck 2018 = Beck et al. (2018); Beck 2019 = Beck et al. (2019); Thompson = Thompson et al. (2019); Proctor = Proctor (2023); Pooled Exp = results from pooling all four experiments.

^2^r = Pearson’s Correlation Coefficient; Cb = bias correction factor; CCC = Lin’s concordance correlation coefficient; RMSE = root mean square error, % of ≥ 3 min visit duration mean.

**Figure 1.**
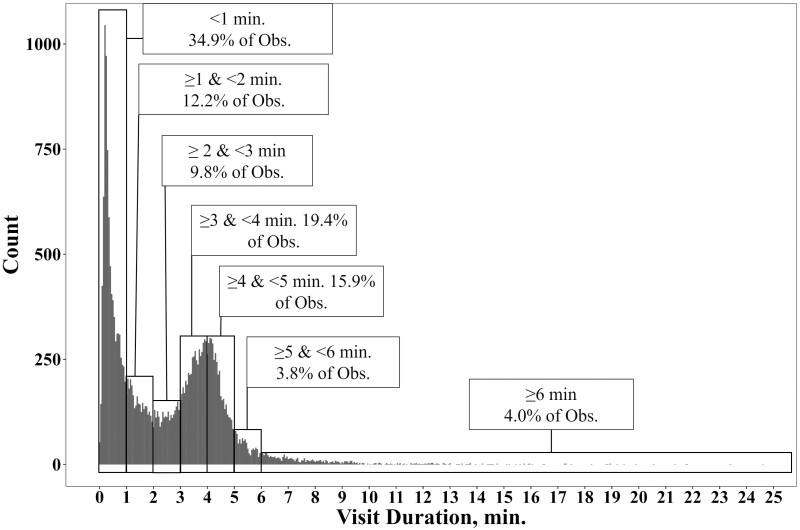
Histogram of visit durations across three experiments which utilized an automated head chamber system (AHCS; GreenFeed; C-Lock Inc., Rapid City, SD). This figure also displays the proportion of total visits that are grouped up by ranges of visit durations. These visits are from four experiments (Beck et al., 2018, 2019; Thompson et al., 2019; Proctor, 2023).

It appears that as visit duration increases, the estimates of CO_2_, O_2_, and CH_4_ also increase, until 3-4 min visit duration, at which time the estimates decrease again (Table 3). Visits less than 1 min, ≥ 1 min and < 2 min, and ≥ 2 min and < 3 min were −24.2%, −11.5%, and −7.3% lower for CO_2_; −44.6%, −12.5%, and −5.4% lower for O_2_; and −69.5%, −22.9%, and −4.9% lower for CH_4_ compared with visits ≥ 3 min in duration, respectively. Estimates of CO_2_, O_2_, and CH_4_ from visits that were ≥ 3 min and < 4 min in duration were 1.4%, 1.1%, and 1.8% greater than the estimates from visits ≥ 3 min, respectively. Finally, the estimates from visits that were ≥ 4 and < 5, ≥ 5 and < 6, and > 6 min were on average less than the estimates from visits ≥ 3 min by 0%, −0.8%, −1.0% for CO_2_; −0.8%, −1.4%, and −6.3% for O_2_; and −1.0%, −3.3%, and −7.7% for CH_4_, respectively. Table 4 reports the r, Cb, CCC, and RMSE for each of the gases and for each of these visit ranges. Figure 1 presents a histogram of visit duration and provides the proportion of total visits that each of these visit duration ranges represents.

**Table 3.** Mean ± the standard deviation of number of visits per animal, visit duration, CO_2_, O_2_, and CH_4_ emissions from data collected by automated head chamber systems (AHCS; GreenFeed, C-Lock Inc., Rapid City, SD) from four different experiments (Beck et al., 2018, 2019; Thompson et al., 2019; Proctor, 2023) as affected by observation (Obs.) duration ranges.

	Visit duration ranges, min.							
Items^1^	Obs. ≥ 3	Obs. < 1	1 ≤ Obs. < 2	2 ≤ Obs. < 3	3 ≤ Obs. < 4	4 ≤ Obs. < 5	5 ≤ Obs. < 6	Obs. ≥ 6
animal	103	103	101	103	103	103	93	92
Visits	101.1 ± 61.26	81.1 ± 91.82	28.9 ± 32.38	22.9 ± 15.75	45.5 ± 17.78	37.4 ± 31.79	9.9 ± 10.21	10.4 ± 17.61
Dur.	4.2 ± 0.54	0.37 ± 0.089	1.4 ± 0.12	2.5 ± 0.11	3.5 ± 0.087	4.4 ± 0.092	5.4 ± 0.14	7.8 ± 1.05
CO_2_	8,208.5 ± 1,908.90	6,224.5 ± 1,113.31	7,261.8 ± 1,320.61	7,610.5 ± 1,372.53	8,324.0 ± 2,014.69	8,209.2 ± 2,009.77	8,178.4 ± 1,856.26	7,790.5 ± 1,855.03
O_2_	5,730.8 ± 1,186.38	3,173.9 ± 718.90	5,012.1 ± 820.63	5,420.1 ± 955.67	5,794.6 ± 1,240.59	5,684.8 ± 1,214.77	5,651.4 ± 1,186.56	5,367.3 ± 11,199.00
CH_4_	164.3 ± 32.42	50.1 ± 21.61	126.6 ± 50.35	156.2 ± 43.08	167.3 ± 30.84	162.7 ± 36.00	158.9 ± 55.56	151.6 ± 49.75

^1^animal = number of animal observations; visits = number of visits to the AHCS per animal; Dur. = visit duration, min.; CO_2_ = carbon dioxide emissions, g/d; O_2_ = oxygen consumption, g/d; CH_4_ = methane emissions, g/d.

**Table 4.** Comparative statistics for CO_2_ emissions, O_2_ consumption, and methane (CH_4_) emissions estimates between the “gold-standard” (average of all visits ≥ 3 min. visit duration displayed in Table 3) and estimates from the automated head chamber (AHCS; GreenFeed, C-Lock Inc., Rapid City, SD) observations (Obs.) visit duration within specified ranges. These values were generated using data from four separate experiments (Beck et al., 2018, 2019; Thompson et al., 2019; Proctor, 2023).

	Visit duration ranges, min.						
Items^1^^,^^2^	Obs. < 1	1 ≤ Obs. < 2	2 ≤ Obs. < 3	3 ≤ Obs. < 4	4 ≤ Obs. < 5	5 ≤ Obs. < 6	Obs. ≥ 6
*CO*_*2*_							
r	0.75	0.83	0.87	0.99	0.99	0.86	0.84
Cb	0.48	0.79	0.89	1.00	1.00	0.99	0.96
CCC	0.36	0.66	0.77	0.99	0.99	0.85	0.80
RMSE	28.82	17.8	14.05	3.49	3.49	11.94	14.28
*O*_*2*_							
r	−0.09	0.65	0.87	0.99	0.99	0.85	0.69
Cb	0.20	0.74	0.94	1.00	1.00	0.98	0.93
CCC	−0.02	0.48	0.82	0.99	0.99	0.83	0.64
RMSE	51.16	20.17	11.51	2.78	3.34	11.53	17.72
*CH*_*4*_							
r	0.52	0.66	0.85	0.96	0.92	0.75	0.66
Cb	0.09	0.65	0.94	0.99	0.99	0.88	0.89
CCC	0.05	0.43	0.79	0.95	0.92	0.66	0.60
RMSE	71.58	32.47	14.95	5.94	8.41	23.19	23.44

^1^CO_2_ = carbon dioxide, g/d; O_2_ = oxygen, g/d; CH_4_ = methane, g/d.

^2^r = Pearson’s correlation coefficient; Cb = bias correction factor; CCC = Lin’s concordance correlation coefficient (Lin, 1989, 2000); RMSE = root mean square error expressed as a percent of the “gold-standard”.

**Figure 2.**
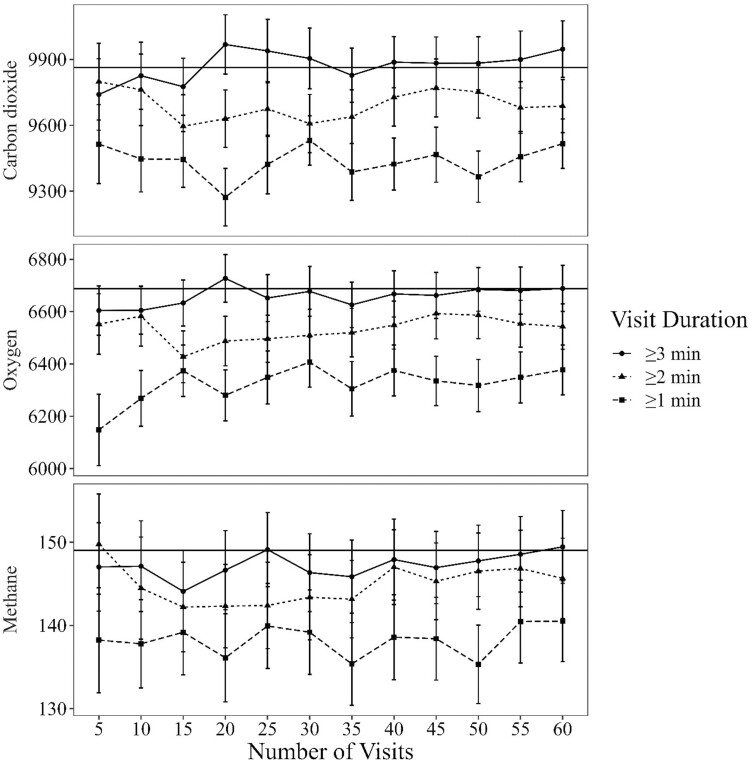
Average carbon dioxide emission, oxygen consumption, and methane emissions estimates (all in g/d) derived from an automated head chamber system (AHCS; GreenFeed, C-Lock Inc., Rapid City, SD). Estimates are three visit duration thresholds (≥ 3 min., ≥ 2 min., or ≥ 1 min.) and with number of visits (5 to 60, increasing by increments of 5) selected at random. The error bars represent the standard error of the mean. The horizontal line represents the average of the “gold-standard” [average of all visits ≥ 3 min. visit duration (144 ± 55.01 min; mean ± standard deviation; *n* = 53 animals)]. Data used in this analysis is from Proctor (2023).

The average CO_2_, O_2_, and CH_4_ flux estimates were always lower than the estimates derived from all visits ≥ 3 min in duration for all number of visits per animal, with a minimum visit duration of ≥ 2 min and ≥ 1 min (Figure 2). Even for the estimates derived from 60 randomly selected animal visit records, the estimates of CO_2_, O_2_, and CH_4_ flux were 1.8%, 2.2%, 2.3% lower for ≥ 2 min visit durations and 3.5%, 4.6%, and 5.7% lower for ≥ 1 min visit durations compared with the estimates derived from all visits ≥ 3 min in duration (the “gold-standard”), respectively. In contrast, the mean estimates were similar for all assigned numbers of visits per animal (5 to 60 visits per animal) with a visit duration of ≥ 3 min compared with the estimates of the “gold-standard”. For instance, the mean differences of 60 visits per animal were < 1% for all gases compared to the “gold-standard”.

**Figure 3.**
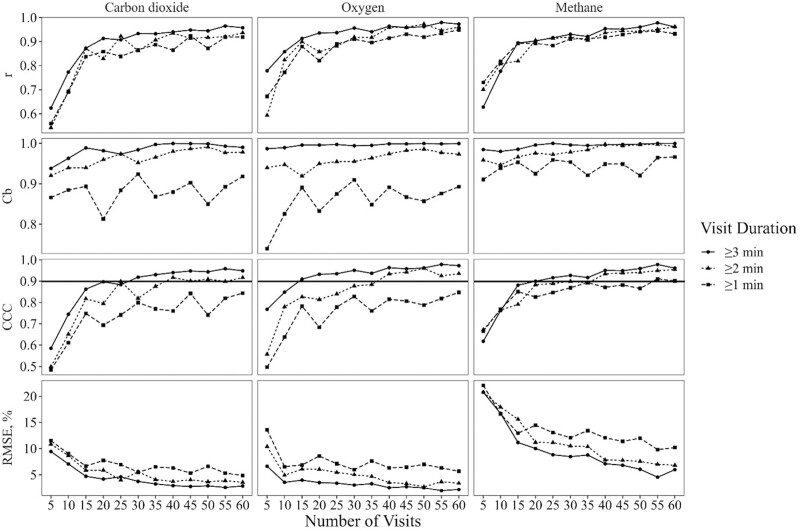
Comparative statistics for carbon dioxide emission, oxygen consumption, and methane emission estimates from an automated head chamber system (AHCS; GreenFeed, C-Lock Inc., Rapid City, SD) between the “gold-standard” [average of all visits ≥ 3 min. visit duration (*n* = 144 ± 55.01; mean ± standard deviation)] and estimates with 3 visit duration thresholds (≥ 3 min., ≥ 2 min., or ≥ 1 min.) and with randomly selected number of visits (5 to 60, increasing by increments of 5). The statistics reported are Pearson’s Correlation Coefficient (r); the bias correction factor (Cb); Lin’s concordance correlation coefficient (CCC; Lin, 1989, 2000); and the root mean square error (RMSE) expressed as a percent of the “gold-standard”. The horizontal line in the CCC graphs represents the 0.90 thresholds set as excellent agreement between the estimates and the “gold-standard”. Data used in this analysis are from Proctor (2023).

The differences between the mean differences of estimates derived from visits ≥ 2 min and ≥ 1 min compared with those estimated from visits ≥ 3 min can be further seen in the Cb of the estimates (Figure 3), where Cb of estimates from visits ≥ 3 min was consistently greater than the Cb of estimates from visits ≥ 2 min and ≥ 1 min. Interestingly, the r of all visit durations appeared to reach a plateau of around 15 to 20 visit records per animal. The number of visits per animal to reach the threshold set (CCC ≥ 0.90) for visits ≥ 3 min in duration was 30 for CO_2_, 15 for O_2_, and 25 for CH_4_. For visits ≥ 2 min, the number of visits per animal needed was 40 for CO_2_, O_2_, and CH_4_. The threshold for the number of visits needed when ≥ 1 min visit duration was used was not reached for CO_2_ and O_2_, but was reached at 55 for CH_4_.

## Discussion

### Visit duration threshold impact on gas flux estimates

The first objective was to determine the effects of utilizing different visit duration thresholds on estimates of CO_2_, O_2_, and CH_4_ from already completed experiments. This would indicate if studies employing different minimum visit durations are comparable. Including visits ≥ 2 min and ≥ 1 min in duration only resulted in minor differences in estimates compared with visits ≥ 3 min in duration for CO_2_, O_2_, and CH_4_. Including all available visits consistently resulted in lower, although only marginally less than, estimates of CO_2_, O_2_, and CH_4_ compared with estimates from visits ≥ 3 min across all four experiments. Furthermore, estimates of CO_2_, O_2_, and CH_4_ from visits ≥ 2 min and ≥ 1 min had excellent agreement (CCC ≥ 0.96) with estimates from visits ≥ 3 min. If one considers this result by itself, then it would suggest that researchers could include AHCS visits ≥ 1 min in duration in their analysis without significant consequences to CO_2_, O_2_, and CH_4_ flux estimates. However, these estimates were also derived from a large number of visits per animal (≥ 64.1 visits that are ≥ 2 min and ≥ 70.5 visits that are ≥ 1 min). Arthur et al. (2017) demonstrated that the mean difference between CH_4_ and CO_2_ estimates derived from ≥ 2 min compared with those from ≥ 3 min diminished when the number of visits per animal increased from 15 to 20 to 50 to 80. This raises the question of what happens to the number of visits required per animal when different minimum visit duration thresholds are utilized? We discuss this question below.

### Visit duration range effects on gas flux estimates

The second objective of this experiment was to explore how the estimates of CO_2_, O_2_, and CH_4_ differ with increasing visit duration lengths. There was a peak for visit durations of around 4-min. (Figure 1). This peak likely occurs because the AHCS feed delivery system is set to dispense feed every 24 to 30 s, with 6 to 8 drops per visit, thereby encouraging animals to remain at the AHCS for around 3 min. All gas flux estimates were lowest for visit duration observations < 1 min, then gradually increased until visit durations ≤ 3 min and < 4 min, but then decreased at longer durations. Furthermore, CCC values were lowest for visits < 1 min (0.36), reached a peak for visits < 4 min and ≥ 3 min (0.99), and for visits < 5 min and ≥ 4 min in duration (0.99). After this peak, CCC values again decreased for longer visit duration ranges (i.e., those ≥ 5 min; CCC ≤ 0.85). However, these lower CCC values were driven by decreased precision (r ≤ 0.86) and not necessarily by accuracy (Cb ≥ 0.96). As these visit ranges had a low number of visits per animal (≤ 10.4), the lower precision for these longer visit durations may be due to a low number of visit observations per animal. To the best of our knowledge, the effects of visit duration range have not been investigated in this manner in previous experiments, making it difficult to compare these results to previous studies. However, based on these results we recommend excluding visit durations < 3 min. Longer visit durations (i.e., ≥ 5 min) had high accuracy, but poorer precision compared to visits ≥ 3 min. Based on the high accuracy of the longer-duration visits, we do not recommend removing these relatively long visits. Furthermore, the poorer precision for visits ≥ 5 min in duration were likely due to the number of visits per animal being relatively few with not all animals being represented in this visit duration range.

### Visit duration threshold impact on number of visits required

The final objective of this experiment was to determine how different visit duration thresholds would influence the number of visits required to achieve adequate estimates of CO_2_, O_2_, and CH_4_ flux. To achieve this objective, only data from Proctor (2023) was utilized, as the majority of steers (*n* = 53 out of 54) used in this investigation had greater than 60 visits (Proctor (2023) had 144 visits per animal on average). Fifty-five visits per animal were required to reach the threshold for adequate agreement set of CCC ≥ 0.90 for CH_4_ and was not achieved for CO_2_ and O_2_ when visits ≥ 1 min were used. As the ≥ 1 min visit duration threshold was deemed inadequate, it is safe to say that visits < 2 min in duration should be excluded. Next, 40 visits per animal were required to achieve the CCC ≥ 0.90 threshold for all gases when visits ≥ 2 min in duration were used. Lastly, when visits ≥ 3 min in duration were used, it required 30 visits per animal for CO_2_, 15 for O_2_, and 25 for CH_4_. As such, we recommend that 30 visits per animal be used as the minimum number of records when the visit duration threshold is set at 3 min. These recommendations fall in line closely with previous recommendations, where Gunter and Beck (2018), in a review of AHCS published literature, reported that most research suggested 30 to 50 animal visits to the AHCS. For example, Arthur et al. (2017) recommended 45 visit records per animal for CH_4_ and CO_2_ estimates and Dressler et al. (2023) recommended 38 visit records per animal for CH_4_ and 40 visit records per animal for CO_2_ and O_2_, when a 2-min visit duration threshold is used. Furthermore, Arthur et al. (2017) recommended that 30 visit records per animal for estimating CO_2_ and O_2_ when a 3-min visit duration threshold is used.

Arthur et al. (2017) recommended setting the visit duration threshold to 3-min. This recommendation was based on estimates from visits ≥ 3 min had greater precision (lower heterogeneous variances) than estimates from visits ≥ 2 min. Furthermore, Arthur et al. (2017) reported that estimates of CH_4_ and CO_2_ were consistently lower when visits ≥ 2 min duration were used than when visits ≥ 3 min duration were used. This closely aligns with the observations of the current experiment. When all visits from the four experiments were grouped into visit ranges (Table 3), the estimates of CO_2_, O_2_, and CH_4_ from visits ≥ 2 min and < 3 were 7.3%, 5.4%, and 4.9% lower than estimates from visits ≥ 3 min. Furthermore, the mean estimates of CO_2_, O_2_, and CH_4_ were consistently lower for CO_2_, O_2_, and CH_4_ when ≥ 2 min visit duration threshold was used compared with a 3 min visit duration threshold (Figure 2). As the values of CO_2_, O_2_, and CH_4_ are lower when visit duration is < 3 min, we recommend removing visits < 3 min in duration.

Based on the number of visits per d observed in the grazing studies with a 3 min minimum visit duration threshold, to achieve the 30 required visits an experiment would need to be conducted for around 19 to 20 d. In confinement, where visits per d is greater, an adequate estimate of CH_4_ could be achieved in 13-d. Furthermore, with a 2 min minimum visit duration threshold, to achieve the 40 required visits, a grazing study would need to be conducted for 22 to 25 d, whereas a confinement study could be conducted in 15 d. This is important information, because based on the visit information presented in Table 1, one could conclude that using 2 min visit duration threshold would increase the number of visits obtained per animal and therefore balance out the increased visit requirements. However, based on the number of visits per day observed in the four experiments utilized in the current analysis, using a 2-min visit threshold would increase the number of experimental days needed to obtain adequate estimates of gas flux by 18.4% on average.

## Conclusions

The current analysis was conducted using data from beef cattle in grazing and confined settings; however, we postulate that these results could be applicable to other production settings. The results of this experiment suggest that the minimum visit duration threshold utilized in AHCS experiments influences the number of visits needed to achieve adequate estimates of CO_2_, O_2_, and CH_4_. The results of this current study suggest that if visits that are ≥ 3 min in duration are utilized, then 30 visits were needed to achieve excellent agreement for CO_2_, O_2_, and CH_4_. If visits that are ≥ 2 min in duration are included, then 40 visits are necessary. It is important to note, that estimates of CO_2_, O_2_, and CH_4_ flux from visits lasting < 3 min and ≥ 2 min were lower than the estimates derived from visits that were ≥ 3 min by −7.3%, −5.4%, and −4.9%, respectively. Therefore, if papers do choose 2 min as the minimum visit duration, it should be recommended that visit frequency by visit length be reported. This would be in line with how most published literature reports visit behavior relative to time of day for meal-fed cattle. Accordingly, we recommend a minimum visit duration of 3 min and a minimum of 30 visit records per animal when using the AHCS to estimate CO_2_, O_2_, and CH_4_. However, if researchers choose to set ≥ 2-min visit duration as their threshold, they should target 40 visit records per animal.

## Supplementary Material

skae158_suppl_Supplementary_Material

skae158_suppl_Supplementary_Data

## Abbreviations

AHCS: automated head chamber system
BW: body weight
Cb: bias correction factor
CCC: Lin’s concordance correlation coefficient
CH4: methane
CO2: carbon dioxide;
O2: oxygen
r: Pearson’s correlation coefficient
RMSE: root mean square error
