# Supplementary material for: Recommendations on visit duration and sample number requirements for an automated head chamber system
Source: J Anim Sci. 2024 Jun 4;102:skae158. doi: 10.1093/jas/skae158 (PMC11190786; doi:10.1093/jas/skae158)
Supplement: skae158_suppl_Supplementary_Material [file skae158_suppl_supplementary_material.docx]

| **Supplementary Table 1.** Comparing gas flux estimates estimated from an automated head chamber system (GreenFeed; C-Lock inc., Rapid City, SD) using an arithmetic mean or time bin averaging approach as suggested by Manafiazar et al. (2017). | | | | | | | | | | |
| --- | --- | --- | --- | --- | --- | --- | --- | --- | --- | --- |
|  | Arithmetic | |  | Time Bin | |  | Comparative Statistics^1^ | | | |
| Item^2^ | Mean | SD |  | Mean | SD |  | r | Cb | CCC | RMSE |
| Beck 2018 |  |  |  |  |  |  |  |  |  |  |
| CO_2_ | 7,143.3 | 564.93 |  | 7,106.6 | 585.91 |  | 0.94 | 1.00 | 0.94 | 2.78 |
| O_2_ | 5,230.6 | 432.51 |  | 5,149.9 | 391.08 |  | 0.97 | 0.97 | 0.95 | 2.46 |
| CH_4_ | 198.3 | 22.54 |  | 211.6 | 26.20 |  | 0.90 | 0.85 | 0.76 | 8.25 |
| Beck 2019 |  |  |  |  |  |  |  |  |  |  |
| CO_2_ | 6,067.5 | 638.43 |  | 6,152.7 | 640.28 |  | 0.98 | 0.99 | 0.97 | 2.65 |
| O_2_ | 4,461.6 | 467.95 |  | 4,512.5 | 473.63 |  | 0.98 | 0.99 | 0.97 | 2.50 |
| CH_4_ | 176.0 | 23.16 |  | 181.4 | 24.50 |  | 0.95 | 0.97 | 0.92 | 5.04 |
| Thompson |  |  |  |  |  |  |  |  |  |  |
| CO_2_ | 6,117.2 | 387.38 |  | 6,208.6 | 409.63 |  | 0.93 | 0.97 | 0.91 | 2.70 |
| O_2_ | 4,390.3 | 325.51 |  | 4,393.8 | 338.28 |  | 0.96 | 1.00 | 0.96 | 2.08 |
| CH_4_ | 169.5 | 13.51 |  | 172.3 | 14.58 |  | 0.90 | 0.98 | 0.88 | 3.92 |
| Proctor |  |  |  |  |  |  |  |  |  |  |
| CO_2_ | 9,860.5 | 835.81 |  | 9,703.3 | 855.22 |  | 0.94 | 0.98 | 0.92 | 3.50 |
| O_2_ | 6,706.4 | 631.08 |  | 6,635.4 | 633.88 |  | 0.97 | 0.99 | 0.97 | 2.37 |
| CH_4_ | 149.9 | 33.00 |  | 140.9 | 29.84 |  | 0.96 | 0.95 | 0.92 | 9.06 |
| Pooled Exp. |  |  |  |  |  |  |  |  |  |  |
| CO_2_ | 8,208.5 | 1,908.9 |  | 8,152.5 | 1,811.60 |  | 0.99 | 1.00 | 0.99 | 3.36 |
| O_2_ | 5,730.8 | 1,186.38 |  | 5,693.7 | 1,148.80 |  | 0.99 | 1.00 | 0.99 | 2.41 |
| CH_4_ | 164.3 | 32.42 |  | 162.8 | 36.71 |  | 0.95 | 0.99 | 0.94 | 7.50 |
| ^1^ r = Pearson’s Correlation Coefficient; Cb = bias correction factor; CCC = Lin’s Concordance Correlation Coefficient; RMSE = Root Mean Square Error as a percent of the “time bin” average.  ^2^ Beck 2018 = Beck et al. (2018); Beck 2019 = Beck et al. (2019); Thompson = Thompson et al. (2019); Proctor = Proctor (2023); Pooled Exp. = data from all 4 experiments were included. | | | | | | | | | | |


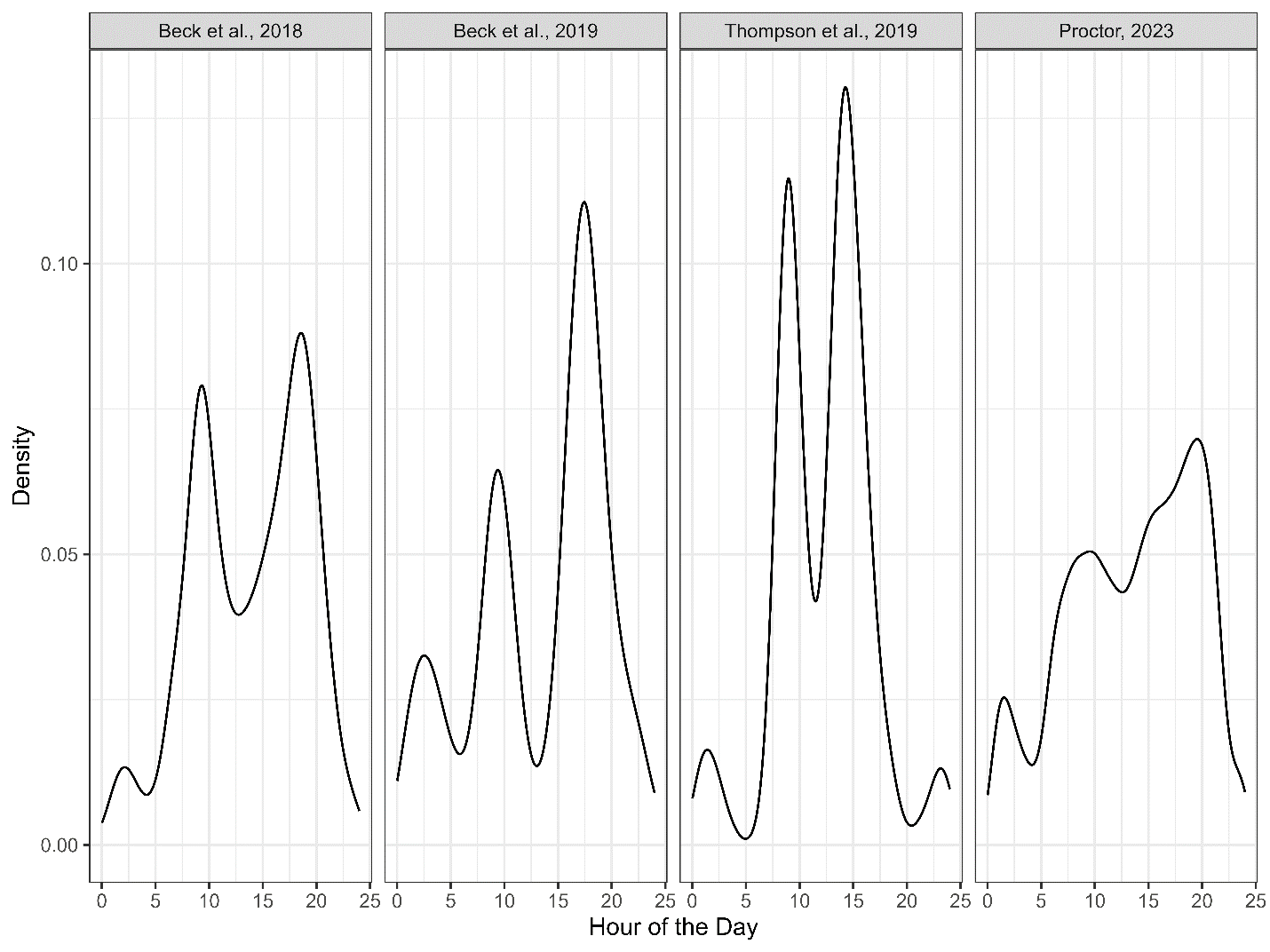


**Supplementary Figure 1.** Density plots for visits across hour of the day for the four experiments used in the current analysis (Beck et al., 2018; Beck et al., 2019; Thompson et al., 2019; Proctor, 2023).
